# Perception of biological motion in visual agnosia

**DOI:** 10.3389/fnbeh.2012.00056

**Published:** 2012-08-28

**Authors:** Elisabeth Huberle, Paul Rupek, Markus Lappe, Hans-Otto Karnath

**Affiliations:** ^1^Section Neuropsychology, Center of Neurology, Hertie-Institute for Clinical Brain Research, University of TübingenTübingen, Germany; ^2^Neurology and Neurorehabilitation Center, State Hospital LucerneLucerne, Switzerland; ^3^Psychological Institute I, Faculty of Psychology, University of MünsterMünster, Germany

**Keywords:** vision, agnosia, ventral stream, biological motion, perception

## Abstract

Over the past 25 years, visual processing has been discussed in the context of the dual stream hypothesis consisting of a ventral (“what”) and a dorsal (“where”) visual information processing pathway. Patients with brain damage of the ventral pathway typically present with signs of visual agnosia, the inability to identify and discriminate objects by visual exploration, but show normal perception of motion perception. A dissociation between the perception of biological motion and non-biological motion has been suggested: perception of biological motion might be impaired when “non-biological” motion perception is intact and vice versa. The impact of object recognition on the perception of biological motion remains unclear. We thus investigated this question in a patient with severe visual agnosia, who showed normal perception of non-biological motion. The data suggested that the patient's perception of biological motion remained largely intact. However, when tested with objects constructed of coherently moving dots (“Shape-from-Motion”), recognition was severely impaired. The results are discussed in the context of possible mechanisms of biological motion perception.

## Introduction

Motion perception is an important requirement for an efficient interaction with our environment. Over the past 25 years, visual processing has been extensively discussed in the context of the dual stream hypothesis (Mishkin and Ungerleider, 1982; Goodale and Milner, 1992): Cortical centres along the ventral (“what”) pathway play a critical role in object recognition and are specialized for the representation of shape information (Malach et al., 1995; Grill-Spector et al., 2001; Lerner et al., 2001; Kourtzi and Kanwisher, 2001; Denys et al., 2004), while areas of the dorsal (“where”) pathway are involved in action-related visual processing and in motion processing (Newsome et al., 1985; Newsome and Pare, 1988; Zeki et al., 1991; Tootell et al., 1995a,b; Schenk and Zihl, 1997). Brain damage of the ventral pathway typically leads to visual agnosia. In particular, visual form agnosia—the inability to discriminate between and recognize simple geometric shapes and objects in the context of intact basic visual abilities such the analysis of contrast and color—has been observed in patients with lesion of the ventromedial occipito-temporal cortex, i.e., the fusiform and lingual gyri (Karnath et al., 2009). However, numerous tasks in daily life require the combined analysis of motion and form. For example, the combined analysis of motion and form is required for the perception of shapes that are defined by coherently moving dots placed in a background of random moving dots (“Shape-from Motion”), which have been associated with an activation of the ventral and dorsal pathway (e.g., Altmann et al., 2004). Another example can be found in the perception of biological motion, which has been linked to an activation of the ventral and dorsal pathway: besides motion sensitive areas of the dorsal pathway and form selective areas of the ventral pathway a specialized network has been proposed for the perception of biological motion (Vaina et al., 2001b; Michels et al., 2005; Vangeneugden et al., 2009) that involves specifically the anterior superior temporal sulcus (STSa) and superior temporal gyrus (STG) predominantly of the right hemisphere (Perrett et al., 1985; Posner and Dehaene, 1994; Grossman et al., 2000; Puce and Perrett, 2003). Patients with brain damage of the STSa and STG might thus present with impaired perception of biological motion (Vaina et al., 2001a,b; Battelli et al., 2003; Akiyama et al., 2006), while the perception of non-biological motion remains normal (Vaina et al., 1990; Cowey and Vaina, 2000). “Point-light walkers”—a well established model to investigate the perception of biological motion—consist of dots that are placed at locations on invisible lines connecting the main joints of arms and legs of a moving body (Johansson, 1975; Beintema and Lappe, 2002; Casile and Giese, 2005; Troje et al., 2006).

The present study aimed to investigate the impact of form analysis for the perception of biological motion in a patient with severe visual agnosia. First, the patient's ability to perceive motion *per se* was tested by presenting the patient with static and moving dot stimuli at different levels of motion coherency. Further, biological motion perception was investigated by applying short movies of typical human whole-body movements and facial movements, that either displayed a human actor or a “point-light walker”.

## Materials and methods

### Subjects

One neurological patient (WH) and four healthy controls (mean age 64 years, range 57–72 years; two males and two females) without a history of brain damage participated in the study. All had normal or corrected-to-normal vision. All subjects gave their informed consent for participation which has been performed in accordance with the ethical standards laid down in the 1964 Declaration of Helsinki.

### Patient WH

WH, a 67-year-old, right-handed man, was admitted to the neurological department with a history of unspecific progressive “visual impairment” for several years affecting his reading abilities, object recognition, face perception and color discrimination. Neurological and ophthalmological examination was normal except for a mild peripheral visual field deficit of the right hemifield. Visual acuity was reduced to 0.2 for the left as well as the right eye. Signs of an involvement of cranial nerves, normal pressure hydrocephalus and moving disorders were not present. Neuropsychological testing revealed severe visual agnosia, prosopagnosia and alexia including the patient's inability to identify any of ten line drawings of common objects (e.g., tree, car, and dog) and failure to recognize any of ten other common objects (e.g., fork, candle, and brush) placed in front of him for an unlimited duration. WH could not read any of ten single words or identify individual letters presented for an unlimited duration. The patient was not able to detect the general context of complex images of the type of the Broken Window Picture from the Stanford Binet Intelligence Test (Binet and Simon, 1905). In addition, severe deficits in color perception were present. In contrast to his severely restricted abilities of object and face recognition, haptic exploration of real objects allowed the patient to correctly identify all of these objects. The patient was able to grasp all of ten common objects presented in front of him with the correct orientation and hand aperture. Signs of spatial neglect were not observed.

**Figure 1:**
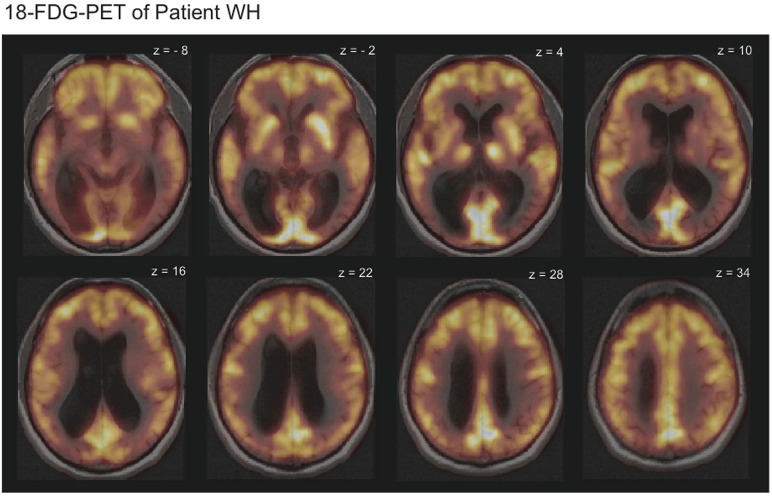
**18-Fluorodeoxyglucose positron emission tomography (FDG-PET-CT) scans overlaid with the magnetic resonance imaging (MRI) scans revealed reduced metabolism in the temporo-occipital cortex bilaterally for patient WH.** Displayed is HW's 18-FDG-uptake in comparison to the average of 20 healthy subjects. Bright yellow colors indicate high and dark red colors low 18-FDG-uptake.

T1- and T2-weighted magnetic resonance imaging of WH showed no signs of focal brain damage. An 18-Fluorodeoxyglucose positron emission tomography (FDG-PET) revealed diffusely reduced metabolism in the temporo-occipital cortex bilaterally in comparison with the data of 20 healthy subjects (Figure 1). Cerebrospinal fluid analysis indicated normal results for ß-amyloid-peptide 1–42 and hyperphosphorylated tau-protein as markers of Alzheimer's disease. Posterior cortical atrophy (Tang-Wai et al., 2004) was diagnosed.

### Visual stimuli, design, and procedure

All experiments were conducted in a darkened environment. In contrast to WH, the healthy control subjects were tested only in Experiments 2 and 3. With respect to our variable of interest (percent of correct responses) we could assume intact performance in the healthy control subjects in Experiment 1. Prior to the presentation of the stimuli, the subjects were familiarized with the type of task and stimuli in a short practice session. For the practice session and Experiment 1–3, the stimuli covered an area of 12.5° × 12.5° with a viewing distance of 50 cm to the monitor. All stimuli were presented in a random order. Each trial was initiated by the experimenter when the subjects attended the center of the monitor placed in front of them and indicated readiness. After the stimulus presentation, the experimenter coded the verbal response given by the subject, which was required for the onset of the next trial.

#### Experiment 1 (“motion identification/discrimination”)

**Figure 2:**
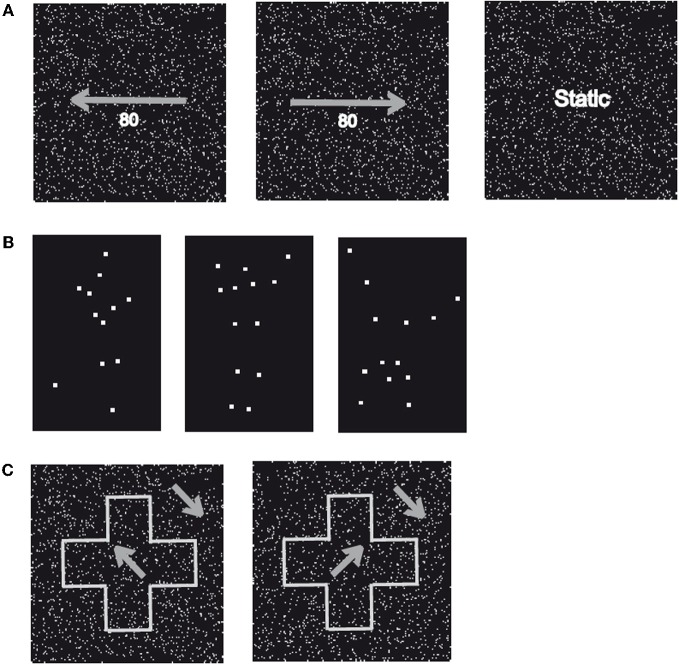
**Sample stimuli. (A)** Displayed are two patterns of randomly moving dots with a motion coherency of 80% toward the left (left) or right (centre). In addition, static patterns of randomly assigned dots (right) with identical stimulus parameters were applied. **(B)** Displayed are three examples (“Running,” “Jumping,” and “Turning a Cartwheel”) of the human movements used in Experiment 2 (“Biological Motion”). Stimuli consisted of white dots placed at locations on (invisible) lines connecting the main joints of upper arm, forearm, upper leg, and lower leg and were presented on a black background (“Point-Light Motion”). **(C)** Displayed is a global shape (“Cross”) that consists of coherently moving dots with a direction of motion rotated 45° counter clockwise (left) or clockwise (right) from the vertical axis presented on a background of coherently moving dots rotated 45° counter clockwise from the vertical axis (“Shape-from Motion”).

First, we aimed to exclude the possibility that WH had a general deficit to perceive low-level motion. In the first part of the experiment (“Motion Identification”, Figure 2A), we presented patterns of moving dots or static images of these dots. The movies were constructed of 120 individual frames, which were presented for 50 msec each resulting in a presentation duration of 6000 msec. The frames consisted of a random pattern of white dots placed in a black background with an average of 9 dots/° visual angle (VA), an individual size of 6 pixels and a mean lifetime of 550 msec before the disappearance of the dots. A random direction of motion was appointed to the dots with an equal distribution of all possible directions (right, left, up, down). In addition, we modulated the coherency of the dots in steps of 20% resulting in five different levels (20%, 40%, 60%, 80%, and 100%), in the way that coherently moving dots either moved to the left or the right. For the static images, we used images of white dots randomly placed in a black background with an average of 9 dots/° VA and a size of 0.1° VA. The same image was presented for 120 frames. Both conditions (“Motion” and “Static”) were repeated 20 times resulting in a total number of 40 trials with an equal number of repetitions in the “Motion” condition for the different levels of motion coherency and motion direction. WH was instructed to indicate if the dot pattern was moving or static.

Subsequently, we tested the patient's ability to discriminate the direction of motion (“Motion Discrimination”) for the movies of moving dots described for the first part of the experiment. The patient was now instructed to indicate the direction of the coherently moving dots (“Left” vs. “Right”). For all levels of motion coherency (20%, 40%, 60%, 80%, and 100%), ten repetitions for each direction of motion were used resulting in a total number of 100 trials.

#### Experiment 2 (“biological motion”)

In the first part of Experiment 2, we tested the subjects' ability to recognize ordinary biological motion (“Full Body Motion”) by presenting six different sequences of human movements (Walking, Jumping, Swimming, Crawling, Turning a Cartwheel, and Playing Soccer). These movements were performed by a human male actor in front of a blue background. The sequences consisted of an average of 174 individual frames (minimum 94 frames, maximum 234 frames) presented for 40 msec resulting in a mean presentation duration of 6960 msec (minimum 3760 msec, maximum 9360 msec) and displayed the same movement twice without delay. The subjects were instructed to name the type of movement for each trial. All movies were repeated 10 times resulting in a total number of 60 trials.

Subsequently, we investigated the subjects' ability to recognize point-light biological motion. The same set of movements (Walking, Jumping, Swimming, Crawling, Turning a Cartwheel, and Playing Soccer) was used but the stimuli were now derived from recordings with a body tracker (Ascencion Motion Star) of the joint positions of a human actor. White dots were placed on each of the main joints of the body (shoulders, elbows, hands, hips, knees, and ankles) and the head, which were extracted by a computer and presented in a black background (“Point-Light Motion,” Figure 2B). These movies were constructed of 100 individual frames presented for 40 msec resulting in a presentation duration of 4000 msec and displayed the same movement twice without delay. In parallel to the first part of Experiment 2, the subjects were instructed to name the movement for each trial. Each movie was repeated 20 times resulting in a total number of 120 trials.

#### Experiment 3 (“shape-from motion”)

In Experiment 3, we tested the subjects' ability to recognize shapes defined by coherently moving dots (“Shape-from Motion”). We presented sequences, which displayed one of six simple geometrical figures (Arrow, Cross, Heart, Moon, Star, and Triangle), that consisted of coherently moving dots with direction of motion either 45° clockwise or counterclockwise from the (invisible) vertical axis, while the direction of motion for the dots of the background was rotated by 90° or 180° clockwise (Figure 2C). Each sequence was constructed of 120 individual frames presented for 50 msec resulting in a presentation duration of 6000 msec. For each frame, we used an average of 55 dots/° VA with an individual size of 0.02° VA, a speed of 0.91° VA/sec for the object as well as the background and a mean lifetime of individual dots of 550 msec before the disappearance of the dots. The subjects were instructed to name the geometrical figure for each trial. All shapes were displayed ten times resulting in a total number of 60 trials.

## Results

We computed the percent of correct responses (CR) for each experiment. Performance above chance level was defined by applying the 95%-confidence interval (95%-CI) for the binomial distribution for each task (“Motion Identification,” “Motion Discrimination,” “Full Body Motion,” “Point-Light Motion,” “Face Motion,” and “Shape-from Motion”).

For the healthy control subjects, perfect performance of 100% CR was observed for Experiment 2 and 3.

### Patient WH

#### Experiment 1 (“motion identification/discrimination”)

WH showed preserved perception of low-level motion. In detail, he showed perfect performance (100% CR) in the first part of Experiment 1 (“Motion Identification”) and could well discriminate the direction of motion in the second part of Experiment 2 (“Motion Discrimination”: 20%-Coherency: 85% CR, 40%-Coherency: 90% CR, 60%-Coherency: 75% CR, 80%-Coherency: 80% CR, 100%-Coherency: 75% CR; 95%-CI: 40.7–59.3%). Differences between the different directions of motion were not observed.

#### Experiment 2 (“biological motion”)

**Figure 3:**
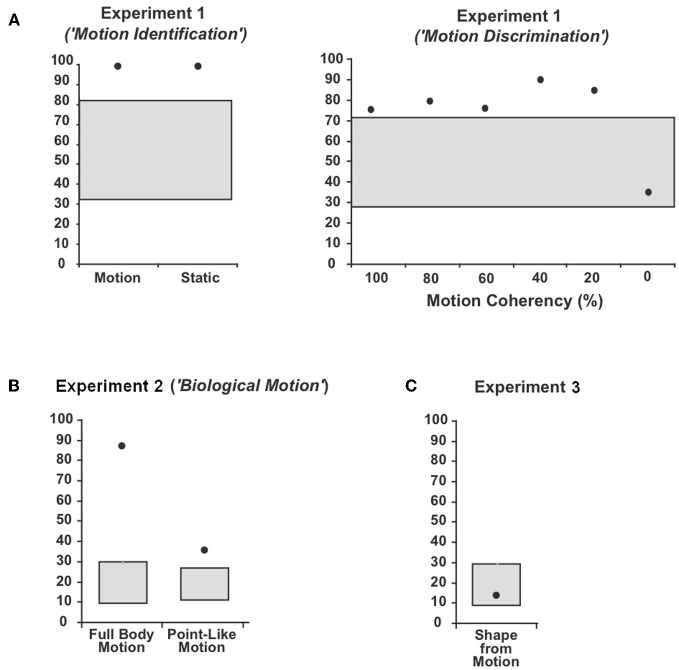
**Average percentage and 95%-confidence interval for the binomial distribution around chance level for patient WH in (A) Experiment 1 (“Motion Identification/ Discrimination”), (B) Experiment 2 (“Biological Motion”) and (C) Experiment 3 (“Shape-from Motion”).** The black dots indicate the average percentage of correct responses (CR) and the grey boxes the 95%-confidence interval for the binomial distribution around chance level.

The patient's ability to identify human full body movements was well preserved (Figure 3) with a performance of 88.3% CR (chance level: 16.6%; 95%-CI: 8.3–28.5%). With 35.8% CR, his ability to identify point-light biological motion significantly exceeded the 16.6%-chance level (95%-CI: 10.5–24.5%) but the low rate of CR indicated difficulties. Differences in the recognition of the different movements were neither observed for the full body movements nor the point-light biological motion.

#### Experiment 3 (“shape-from motion”)

WH showed disturbed shape recognition for shapes defined by coherently moving dots. His performance of 13.3% CR did not exceed the chance level of 16.3% (95%-CI: 8.3–28.5%).

## Discussion

Processing biological motion—human as well as animal motion—has been linked to activation of the ventral as well as dorsal pathway as well as specialized areas in the STS and STGa (Vaina et al., 2001b; Michels et al., 2005; Vangeneugden et al., 2009). It has been reported that the perception of biological motion might be impaired while general motion processing and object recognition remain intact (Vaina et al., 1990). The present study addressed the impact of form analysis for the perception of biological motion in the context of severe visual agnosia. We first demonstrated that our patient shows intact processing of basic (non-biological) motion information such that he was well able to discriminate motion from static information and identify the direction of coherently moving dot patterns. When we tested the patient's ability to recognize biological motion, performance was well above chance level. WH's performance for full body biological motion was close to normal with 88.3% CR, while his ability to identify actions from point-light displays was reduced. The presentation duration was 6000 msec for Experiment 1 and 3, while a mean presentation duration of 6960 msec (min 3760 msec, max 9360 msec) was used for the first part of Experiment 2 (“Full Body Motion”) and 4000 msec for the second part of Experiment 2 (“Point-Light Motion”). Although the presentation duration was identical for Experiment 1 and 3, large differences in the performance were observed with intact motion identification and discrimination in Experiment 1 and disturbed recognition of shapes defined by coherently moving dots in Experiment 3. In addition, differences between the different movements presented in the first part of Experiment 2 were not observed, although the presentation duration largely varied between the movements. Finally, the longer presentation duration of Experiment 3 compared to the second part of Experiment 2 did not facilitate recognition performance. We thus argue that the presentation duration did not have an effect on the recognition performance. The present data rather indicate that the perception of biological motion might remain unaffected in the case of disturbed object recognition. However, form analysis appears to facilitates the perception of biological motion.

“What are mechanisms that mediate the perception of biological motion?” Several functional approaches have been proposed to mediate the recognition of point-light biological motion. These models have focused either on the use of local opponent motion patterns (Giese and Poggio, 2003) or motion trajectories of individual dots (Troje and Westhoff, 2006) and body form information^1^ (Lange and Lappe, 2006). The use of local opponent motion patterns and motion trajectories focuses on the processing of motion rather than shape information and should thus be intact in patients with visual agnosia. Both mechanisms predict normal performance not only for full body biological motion, but also for point-light stimuli. In contrast, the processing of point-light biological motion might rather rely on the integration of individual points into a body form, which is implemented in theories of body form information (Lange and Lappe, 2006). While many aspects of biological motion perception focus on the organization of multiple trajectories into a holistic body form, the perception of single trajectories or the partial representation of a “diagnostic” trajectory that is particularly informative (e.g., feet for walking recognition) might be sufficient (Troje and Westhoff, 2006; Chang and Troje, 2009). WH's residual ability to identify point-light biological motion might thus reflect the use of such individual motion signals that allowed him to perform our categorization task by regarding the global motion patterns or “diagnostic” motion trajectories. The model further assumes a link between the processing of motion and shape information and predicts reduced performance following brain damage of the ventral pathway. WH's high recognition performance for whole-body displays might thus be explained by some residual processing of shape information. However, the differences in the performance between full-body and point-light biological motion indicate that mechanisms of non-biological motion processing have a limited contribution to our findings: similar motion information was used for both types of stimuli (Johansson, 1975). The critical difference between the full body and point-light stimuli can rather be found in the amount of form information, which was high for the whole-body stimuli but reduced (though not fully absent) for the point-light stimuli. Intuitively, HW's difficulties in identifying different types of biological motion correspond roughly to the level of “stimulus abstraction” of the stimuli, which was higher for the point-light stimuli than the full body displays. The patient's ability to recognize shapes defined by coherently moving dots (“Shape-from Motion”) was severely impaired and his performance did not exceed chance level. It appears that HW's residual capacity to process shape information was not sufficient to allow the patient the perception of “Shape-from Motion” stimuli. A reason for the differences in performance between the perception of point-light biological motion and “Shape-from Motion” stimuli might be found in the significance of the specialized network for the perception of biological motion that does not contribute to the perception of shapes derived by motion cues.

Besides more functionally oriented approaches, anatomical considerations should thus be taken into account to explain the present findings. Evidence for a cortical specialization for the perception of biological motion came from findings in children with Williams syndrome, a rare genetic disorder, which presented with impaired motion perception in the context of normal perception of biological motion (Jordan et al., 2002). In contrast to these patients, HW not only showed intact perception of biological motion, but also motion perception. Improved recognition performance for biological motion stimuli might thus be explained if human action recognition relies on specialized mechanisms for the processing of biological motion that is distinct from general form and motion processing. The anterior STSa and STG predominantly of the right hemisphere have been discussed to play a critical role for the perception of biological motion in primates and humans (Perrett et al., 1985; Posner and Dehaene, 1994; Grossman et al., 2000; Puce and Perrett, 2003). In line are observations in patients with brain damage of the STSa and STG or the parietal cortex, who show impaired perception of biological motion (Vaina et al., 2001a,b; Battelli et al., 2003; Akiyama et al., 2006), while their perception of non-biological motion remained unaffected (Vaina et al., 1990; Cowey and Vaina, 2000). HW not only showed intact motion perception, but also normal perception of biological motion for full body movements and only partially impaired performance for point-light biological motion. A well documented case was presented by Vaina and colleagues (2002) who reported a patient with a right hemispheric lesion of the occipito-temporal cortex leading to deficits in local and global motion processing but not the perception of biological motion. Besides a significant contribution of the STG, the voxel-based lesion analysis of a larger group of stroke patients with deficits in the perception of biological motion (Saygin, 2007) and healthy individuals (Saygin et al., 2004) indicated a contribution of premotor frontal areas. Recent data acquired from a larger number of stroke patients with different types of motion processing deficits emphasized a dissociation between basic motion processing and processing of complex motion. They further strengthened the role of the temporal, parietal, and frontal lobe for the perception of biological motion (Billino et al., 2009). HW's limited perception of point-light biological motion might thus be the result of a dysfunction of cortical areas involved in the processing of biological motion, especially the STSa and STG.

Finally, we can not rule out the possibility that the present data underlies a general impairment for the perception of “high-level” motion for which the integration across space and time might be essential. We can also only speculate about the question, if the HW would have been able to recognize the type of biological motion from static images. The patient's inability to recognize facial expressions on static images during standard neuropsychological testing might suggest limited capacities. Unfortunately, HW was not available for further investigation.

In conclusion, our present findings support models of an anatomical specialization for the processing of biological motion and emphasize the role of form analysis as a requirement for the perception of biological motion.

### Conflict of interest statement

The authors declare that the research was conducted in the absence of any commercial or financial relationships that could be construed as a potential conflict of interest.

## Footnotes

^1^Note that although the model of Giese and Poggio (2003) requires the form pathway to action recognition, point-light stimuli have a small impact on the ventral pathway in their model, and therefore, the processing of such stimuli is rather based on local motion signals (see Figure 5 and associated text in Giese and Poggio, 2003).

## References

[B1] Akiyama T. Kato M. Muramatsu T. Saito F. Umeda S. Kashima H. (2006). Gaze but not arrows: a dissociative impairment after right superior temporal gyrus damage. Neuropsychologia 44, 1804–1810 10.1016/j.neuropsychologia.2006.03.007 16616939

[B2] Altmann C. F. Deubelius A. Kourtzi Z. (2004). Shape saliency modulates contextual processing in the human lateral occipital complex. J. Cogn. Neurosci. 16, 794–804 10.1162/089892904970825 15200707

[B3] Battelli L. Cavanagh P. Thronton I. M. (2003). Perception of biological motion in parietal patients. Neuropsychologia 41, 1808–1816 10.1016/S0028-3932(03)00182-9 14527544

[B4] Beintema J. A. Lappe M. (2002). Perception of biological motion without local image motion. Proc. Natl. Acad. Sci. U.S.A. 99, 5661–5663 10.1073/pnas.082483699 11960019 PMC122827

[B5] Billino J. Braun D. I. Böhm K. D. Bremmer F. Gegenfurtner K. R. (2009). Cortical networks for motion processing: effects of focal brain lesions onf perception of different motion types. Neuropsychologia. 47, 2133–2144 10.1016/j.neuropsychologia.2009.04.005 19375433

[B6] Binet A. Simon T. (1905). Methodes nouvelles pour le diagnostic du niveau intellectual des anormaux. Annee Psychol. 11, 191–337

[B7] Casile A. Giese M. A. (2005). Critical features for the recognition of biological motion. J. Vis. 5, 348–360 10.1167/5.4.6 15929657

[B8] Chang D. H. F. Troje N. F. (2009). Characterizing global and local mechanisms in biological motion perception. J. Vis. 9, 1–10 10.1167/9.5.8 19757886

[B9] Cowey A. Vaina L. M. (2000). Blindness to form from motion despite intact static form perception and motion detection. Neuropsychologia 38, 566–578 10689034 10.1016/s0028-3932(99)00117-7

[B10] Denys K. Vanduffel W. Fize D. Nelissen K. Peuskens H. Van Essen D. (2004). The processing of visual shape in the cerebral cortex of human and nonhuman primates: a functional magnetic resonance imaging study. J. Neurosci. 24, 2551–2565 10.1523/JNEUROSCI.3569-03.2004 15014131 PMC6729498

[B11] Giese M. Poggio T. (2003). Neural mechanisms for the perception of biological motion. Nat. Rev. Neurosci. 4, 179–192 10.1038/nrn1057 12612631

[B12] Goodale M. A. Milner A. D. (1992). Separate visual pathways for perception and action. Trends Neurosci. 15, 20–25 1374953 10.1016/0166-2236(92)90344-8

[B13] Grill-Spector K. Kourtzi Z. Kanwisher N. (2001). The lateral occipital complex and its role in object recognition. Vision Res. 41, 1409–1422 10.1016/S0042-6989(01)00073-6 11322983

[B15] Grossman E. Donnelly M. Price R. Pickens D. Morgan V. Neighbor G. (2000). Brain areas involved in perception of biological motion. J. Cogn. Neurosci. 12, 711–720 11054914 10.1162/089892900562417

[B17] Johansson G. (1975). Visual motion perception. Sci. Am. 232, 76–88 1145169 10.1038/scientificamerican0675-76

[B18] Jordan H. Reiss J. E. Hoffman J. E. Landau B. (2002). Intact perception of biological motion in the face of profound spatial deficits: Williams syndrome. Psychol. Sci. 13, 162–167 11934001 10.1111/1467-9280.00429

[B19] Karnath H. O. Rüter J. Mandler A. Himmelbach M. (2009). The anatomy of object recognition–visual form agnosia caused by medial occipitotemporal stroke. J. Neurosci. 29, 5854–5862 10.1523/JNEUROSCI.5192-08.2009 19420252 PMC6665227

[B21] Kourtzi Z. Kanwisher N. (2001). Representation of perceived object shape by the human lateral occipital complex. Science 293, 1506–1509 10.1126/science.1061133 11520991

[B23] Lange J. Lappe M. (2006). A model of biological motion perception from configural form cues. J. Neurosci. 26, 2894–2906 10.1523/JNEUROSCI.4915-05.2006 16540566 PMC6673973

[B24] Lerner Y. Hendler T. Ben-Bashat D. Harel M. Malach R. (2001). A hierarchical axis of object processing stages in the human visual cortex. Cereb. Cortex 11, 287–297 11278192 10.1093/cercor/11.4.287

[B25] Malach R. Reppas J. B. Benson R. R. Kwong K. K. Jiang H. Kennedy W. A. (1995). Object-related activity revealed by functional magnetic resonance imaging in human occipital cortex. Proc. Natl. Acad. Sci. U.S.A. 92, 8135–8139 7667258 10.1073/pnas.92.18.8135 PMC41110

[B26] Michels L. Lappe M. Vaina L. M. (2005). Visual areas involved in the perception of human movement from dynamic form analysis. Neuroreport 16, 1037–1041 15973144 10.1097/00001756-200507130-00002

[B27] Mishkin M. Ungerleider L. G. (1982). Contribution of striate inputs to the visuospatial functions of parieto-preoccipital cortex in monkeys. Behav. Brain Res. 6, 57–77 10.1016/0166-4328(82)90081-X 7126325

[B28] Newsome W. T. Pare E. B. (1988). A selective impairment of motion perception following lesions of the middle temporal visual area (MT). J. Neurosci. 8, 2201–2211 3385495 10.1523/JNEUROSCI.08-06-02201.1988 PMC6569328

[B29] Newsome W. T. Wurtz R. H. Dursteler M. R. Mikami A. (1985). Deficits in visual motion processing following ibotenic acid lesions of the middle temporal visual area of the macaque monkey. J. Neurosci. 5, 825–840 3973698 10.1523/JNEUROSCI.05-03-00825.1985 PMC6565029

[B32] Perrett D. I. Smith P. A. Mistlin A. J. Chitty A. J. Head A. S. Potter D. D. (1985). Visual analysis of body movements by neurones in the temporal cortex of the macaque monkey: a preliminary report. Behav. Brain Res. 16, 153–170 4041214 10.1016/0166-4328(85)90089-0

[B33] Posner M. I. Dehaene S. (1994). Attentional networks. Trends Neurosci. 17, 75–79 7512772 10.1016/0166-2236(94)90078-7

[B34] Puce A. Perrett D. (2003). Electrophysiology and brain imaging of biological motion. Philos. Trans. R. Soc. Lond. B Biol. Sci. 358, 435–445 10.1098/rstb.2002.1221 12689371 PMC1693130

[B36] Saygin A. P. (2007). Superior temporal and premotor brain areas necessary for biological motion perception. Brain 130, 2452–2461 10.1093/brain/awm162 17660183

[B37] Saygin A. P. Wilson S. M. Hagler D. J. Jr. Bates E. Sereno M. I. (2004). Point-light biological motion perception activates human premotor cortex. J. Neurosci. 24, 6181–6188 10.1523/JNEUROSCI.0504-04.2004 15240810 PMC6729669

[B38] Schenk T. Zihl J. (1997). Visual motion perception after brain damage: II. Deficits in form-from-motion perception. Neuropsychologia 35, 1299–1310 10.1016/S0028-3932(97)00005-5 9364499

[B40] Tang-Wai D. F. Knopman D. S. Geda Y. E. Edland S. D. Smith G. E. Ivnik R. J. Tangalos E. G. Boeve B. F. Petersen R. C. (2004). Clinical, genetic, and neuropathologic characteristics of posterior cortical atrophy. Neurology 63, 1168–1174 15477533 10.1212/01.wnl.0000140289.18472.15

[B41] Tootell R. B. Reppas J. B. Dale A. M. Look R. B. Sereno M. I. Malach R. (1995a). Visual motion aftereffect in human cortical area MT revealed by functional magnetic resonance imaging. Nature 375, 139–141 10.1038/375139a0 7753168

[B42] Tootell R. B. Reppas J. B. Kwong K. K. Malach R. Born R. T. Brady T. J. (1995b). Functional analysis of human MT and related visual cortical areas using magnetic resonance imaging. J. Neurosci. 15, 3215–3230 7722658 10.1523/JNEUROSCI.15-04-03215.1995 PMC6577785

[B43] Troje N. F. Sadr J. Geyer H. Nakayama K. (2006). Adaptation aftereffects in the perception of gender from biological motion. J. Vis. 6, 850–857 10.1167/6.8.7 16895463

[B44] Troje N. F. Westhoff C. (2006). The inversion effect in biological motion perception: evidence for a “life detector”? Curr. Biol. 16, 821–824 10.1016/j.cub.2006.03.022 16631591

[B45] Vaina L. M. Cowey A. Eskew R. T. Jr. LeMay M. Kemper T. (2001a). Regional cerebral correlates of global motion perception: evidence from unilateral cerebral brain damage. Brain 124, 310–321 10.1093/brain/124.2.310 11157558

[B46] Vaina L. M. Solomon J. Chowdhury S. Sinha P. Belliveau J. W. (2001b). Functional neuroanatomy of biological motion perception in humans. Proc. Natl. Acad. Sci. U.S.A. 98, 11656–11661 10.1073/pnas.191374198 11553776 PMC58785

[B47] Vaina L. M. Cowey A. LeMay M. Bienfang D. C. Kikinis R. (2002). Visual deficits in a patient with ‘kaleidoscopic disintegration of the visual world’. Eur. J. Neurol. 9, 463–477 10.1046/j.1468-1331.2002.00435.x 12220377

[B48] Vaina L. M. Lemay M. Bienfang D. C. Choi A. Y. Nakayama K. (1990). Intact “biological motion” and “structure from motion” perception in a patient with impaired motion mechanisms: a case study. Vis. Neurosci. 5, 353–369 2265150 10.1017/s0952523800000444

[B49] Vangeneugden J. Pollick F. Vogels R. (2009). Functional differentiation of macaque visual temporal cortical neurons using a parametric action space. Cereb. Cortex 19, 593–611 10.1093/cercor/bhn109 18632741

[B50] Zeki S. Watson J. D. Lueck C. J. Friston K. J. Kennard C. Frackowiak R. S. (1991). A direct demonstration of functional specialization in human visual cortex. J. Neurosci. 11, 641–649 2002358 10.1523/JNEUROSCI.11-03-00641.1991 PMC6575357

